# Additional partial hepatectomy at the time of portal vein ligation accelerates the regeneration of the future liver remnant

**DOI:** 10.1038/s41598-021-90819-x

**Published:** 2021-06-03

**Authors:** Chuanfeng Hua, Weiwei Wei, Tianjiao Zhang, Fengming Xu, Olaf Dirsch, André Homeyer, Utz Settmacher, Uta Dahmen

**Affiliations:** 1 Department of General, Visceral and Vascular Surgery, Jena University Hospital, Jena, Germany; 2 Department of Visceral-, Transplant-, Thoracic- and Vascular Surgery, University Clinic Leipzig, Liebigstraße 20, Haus 4, 04103 Leipzig, Germany; 3 Department of Radiotherapy and Radiation Oncology, Jena University Hospital, Jena, Germany; 4 Institute of Pathology, Klinikum Chemnitz gGmbH, Chemnitz, Germany; 5 Fraunhofer Institute for Digital Medicine MEVIS, Bremen, Germany

**Keywords:** Experimental models of disease, Hepatic portal vein, Hepatocytes

## Abstract

Portal vein ligation (PVL) has been adopted to induce hypertrophy of the future liver remnant (FLR) in patients with primarily irresectable liver tumor. However, regeneration of the FLR is not always sufficient to allow curative resection of the portally-deprived tumor-bearing liver lobe. We hypothesize that simultaneous hepatectomy (PHx) and PVL augments regeneration of the FLR and that the effect is related to the extent of the additional resection. Seventy-two Lewis rats were enrolled into 3 groups: 20%PVL + 70%PHx; 70%PVL + 20%PHx; 90%PVL. Animals were observed for 1, 2, 3 and 7 days postoperatively (n = 6/time point). Liver enzymes, caudate liver/body-weight-ratio, BrdU-proliferation-index (PI), proliferating-cell-nuclear-antigen (PCNA)-mRNA-expression level and autophagy-related-proteins were evaluated. Compared with 90% PVL, additional PHx induced significantly more hypertrophy during the observation time, which was confirmed by significantly higher PI and higher level of PCNA-mRNA expression. Similarly, the additional PHx induced more autophagy in the FLR compared with PVL alone. However, both effects were not clearly related to the extent of additional resection. Additional resection augmented liver regeneration and autophagy substantially compared with PVL alone. Therefore, we concluded that autophagy might play a critical role in regulating hepatocyte proliferation and the size of the FLR after simultaneous PVL + PHx.

## Introduction

The liver is the primary site of metastasis for many tumors, especially of colorectal cancer. Surgical resection is the only curative treatment for malignant liver tumors and offers the patients a chance for long-term survival^1^. At the time of diagnosis, the majority of patients have multiple metastases^2^. High metastatic burden requires extended liver resection. An important risk of extended liver resection is the inadequate size and function of the FLR, which is associated with substantial postoperative morbidity and mortality^3,4^. Therefore, patients with a high tumor load are often excluded from this potentially life-saving therapeutic option^5^.

In the case of initially irresectable liver tumor, different staged procedures were introduced in the clinic: portal vein occlusion followed by PHx, two sequential hepatectomies and the combination of portal vein occlusion and two sequential hepatectomies.

Portal vein occlusion prior to performing the major liver resection is used most frequently to induce hypertrophy of the FLR. Two main technologies were established: portal vein ligation (PVL) and portal vein embolization (PVE)^6,7^. Portal deprivation causes atrophy of the deportalized liver lobe and compensatory hypertrophy of the portally supplied contralateral lobe. The first successful clinical application of this concept was reported by Makuuchi et al.^8^, who performed PVE of the tumor-bearing lobe prior to major hepatectomy. They demonstrated that the technique was feasible and decreased the post-hepatectomy liver failure.

Performing portal vein occlusion prior to the major resection broadens the indications of surgical treatment and is now adopted as one standard procedure for patients with initially irresectable tumors. However, around one-third of the patients undergoing portal vein occlusion in the first stage cannot be subjected to the second stage procedure^9,10^. The most frequently reported reasons are insufficient hypertrophy of the FLR and progression of tumor growth^10^. Portal vein occlusion prior to major hepatectomy can not only induce hypertrophy in the FLR, but also stimulates tumor growth in both, the occluded lobe and the non-occluded lobe^11,12^. It was reported that the growth rate of the tumor tissue in the non-ligated liver lobe may even be higher than the growth rate of the healthy non-tumor liver tissue, resulting in persistent irresectability^13,14^. Thus preoperative portal vein occlusion should not be performed in patients with initially irresectable multiple bilateral colorectal liver metastases.

Another procedure to increase the number of patients eligible for extended surgical resection was introduced by Adam and colleagues, who performed “two-stage (sequential) hepatectomy”^2^. The procedure consisted of two sequential liver resections to remove initially irresectable multiple bilateral liver tumors (MBLBs). Later, two-sequential hepatectomy was combined with portal vein occlusion^15,16^. Jaeke et al. reported a small series of successful two-stage hepatectomy combined with PVE in patients with initially irresectable MBLMs. The first-stage hepatectomy cleared all the tumors located in the FLR (the left lobe) and followed by PVE of the right lobe to induce hypertrophy of the FLR. The second-stage major hepatectomy was performed when the hypertrophy of the FLR was sufficient^15^. Thus the tumor burden was reduced by “cleaning” the FLR completely in the first stage hepatectomy. Using this modified strategy allowed to expand the indications for resecting multiple bilateral colorectal liver metastases and provided selected patients with a chance of a curative treatment. Therefore it is of interest to better understand the effect of combined portal vein occlusion and PHx on intrahepatic size regulation.

In our previous studies^17,18^, we established a rat model combined PVL and PHx. We investigated the influence of additional PHx on the ligated liver lobe. We found that additional PHx abrogated atrophy and induced mild hepatocyte proliferation in the ligated liver lobe^17^. Performing a large additional resection (70%PHx) caused a slight increase in the size of the ligated liver lobe which normally undergoes substantial atrophy. However, performing a small additional resection (20%PHx) did not fully prevent atrophy of the ligated liver lobe, but reduced the extent of atrophy substantially compared to PVL alone. Furthermore, we observed an induction of proliferation, down-regulation of apoptosis and an up-regulation of autophagy in the ligated lobe^18^. In other words, the additional resection had a substantial impact on the size regulation of the ligated lobe, which was related to the extent of the additional resection.

The aim of the present study is to investigate the influence of the additional resection on the size regulation of the non-ligated lobe. We wanted to explore the amount of hypertrophy of the non-ligated FLR after simultaneous PVL and PHx of different extent compared with PVL alone. Furthermore, we wanted to figure out whether the extent of resection itself or the resulting different degree of atrophy in the ligated lobe caused by the different extent of additional liver resection would also affect the extent of hypertrophy in FLR. We were also interested in exploring the role of autophagy in size regulation of the FLR.

We hypothesized that combining PVL and PHx augments the regenerative response in an “extent of resection-dependent” manner compared to PVL alone, possibly facilitated by the induction of autophagy in the FLR.

## Results

To investigate this hypothesis we designed a study with three experimental groups: (I) major PVL alone representing the conventional first step in two-stage hepatectomy without atypical resection; (II) major PVL with additional minor liver mass resection, similar to minor “cherry picking” resection; (III) minor PVL with additional major liver mass resection, mimicking a substantial liver resection at the time of portal vein occlusion. The impact of additional liver resection on augmenting the regeneration of the FLR was compared with conventional simple PVL. Furthermore, the effect of performing different extent of additional resection on autophagy in the FLR was evaluated.

### Additional PHx did not cause more injury compared to PVL alone

**Figure 1:**
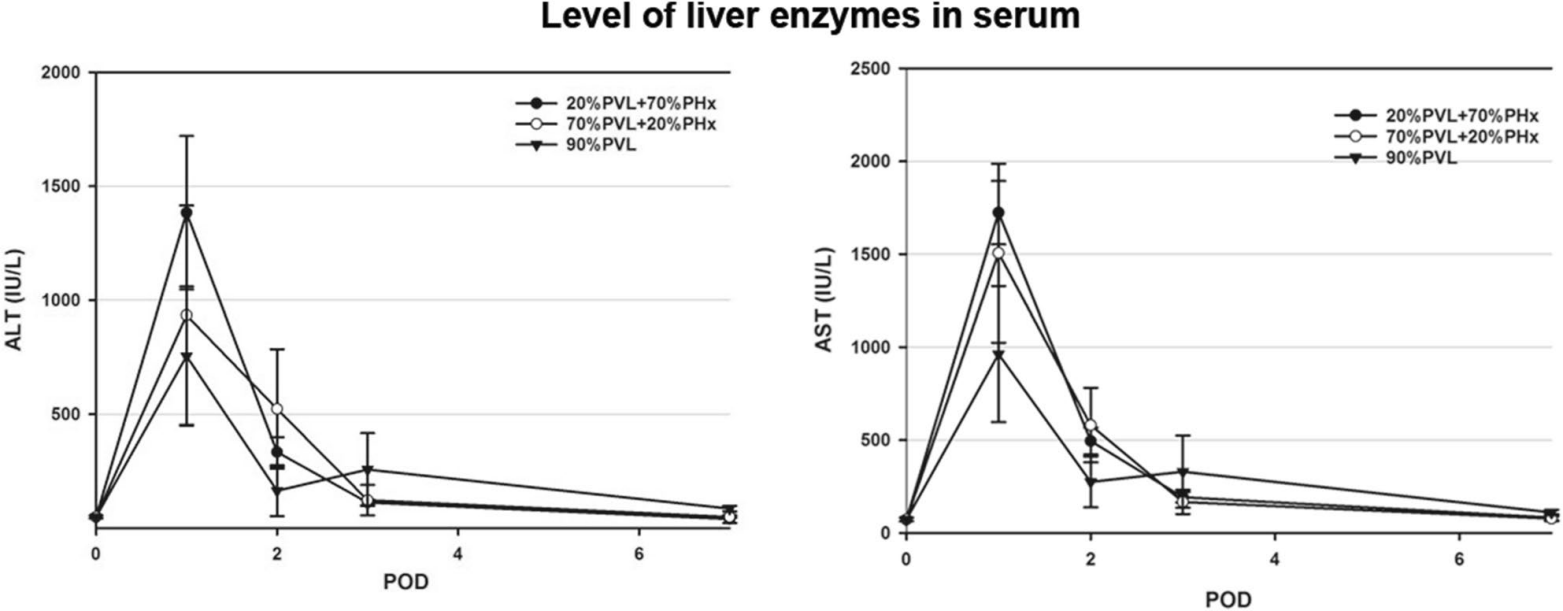
Serum level of ALT and AST from serum sample after simultaneous PVL + PHx and PVL alone. To evaluate the surgical stress and liver injury, the level of serum ALT and AST was measured. The serum ALT and AST were increased rapidly after simultaneous PVL + PHx and PVL alone and decreased to normal level within 7 days. No significant difference was observed between simultaneous PVL + PHx and PVL alone.

As a first step, we assessed whether the additional resection aggravated the damage to the liver compared with extended PVL. We evaluated the postoperative liver enzyme release. Serum alanine aminotransferase (ALT) and aspartate aminotransferase (AST) were selected as markers of acute liver injury following surgical treatment. As partly reported in our previous study^17,18^, additional PHx did not induce significantly more injury to the liver compared with 90% extended PVL. The level of serum ALT and AST peaked on postoperative day (POD) 1 and decreased progressively to normal level on POD7. No significant differences were observed in the level of AST and ALT after additional PHx and 90%PVL alone (Fig. 1).

### Additional PHx induced more hypertrophy in the FLR compared with 90%PVL alone

**Figure 2:**
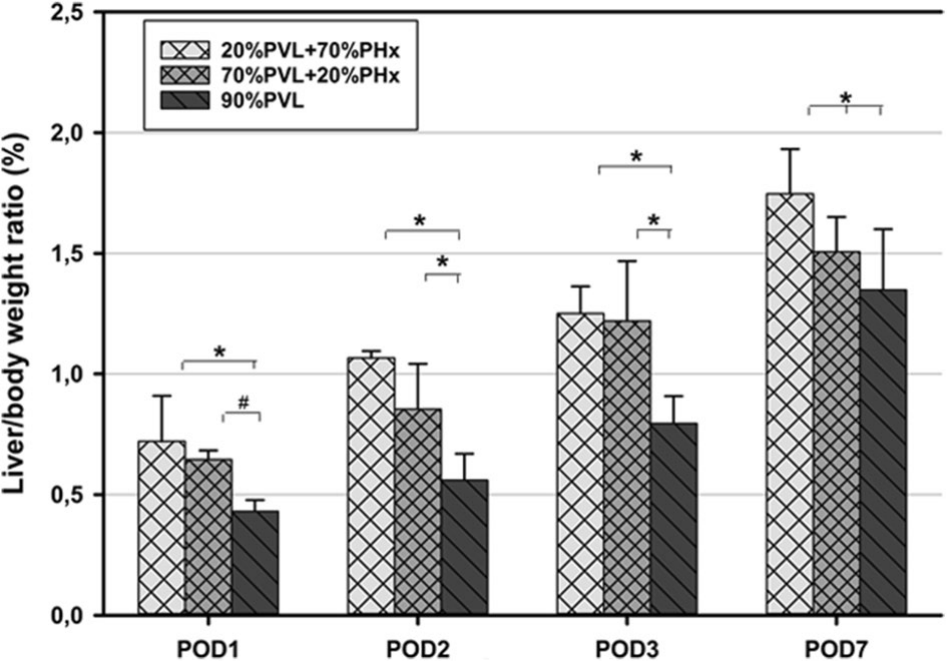
Liver weight to body weght ratio after simultaneous PVL + PHx and PVL alone. The liver/body weight ratio increased steadily after operation. The ratio was significantly higher after additional PHx compared with PVL alone (*p value < 0.05, ^**#**^*p* value < 0.01).

Second, we assessed the hypertrophy of the FLR. To determine the extent of hypertrophy of the non-ligated liver lobe, the caudate lobe to body weight ratio was calculated. At all observation time points, the caudate liver lobe to body weight ratio in the non-ligated remnant liver was significantly higher after simultaneous PVL and PHx compared to PVL alone as shown in Fig. 2 (*p* < 0.05). On POD2, the caudate liver lobe to body weight ratio after additional large resection was even about twofold higher than after PVL alone. However, no significant difference in the size of the caudated lobe (FLR) was observed when comparing large and small additional resection.

### Additional PHx induced more hepatocyte proliferation in the FLR than PVL alone

**Figure 3:**
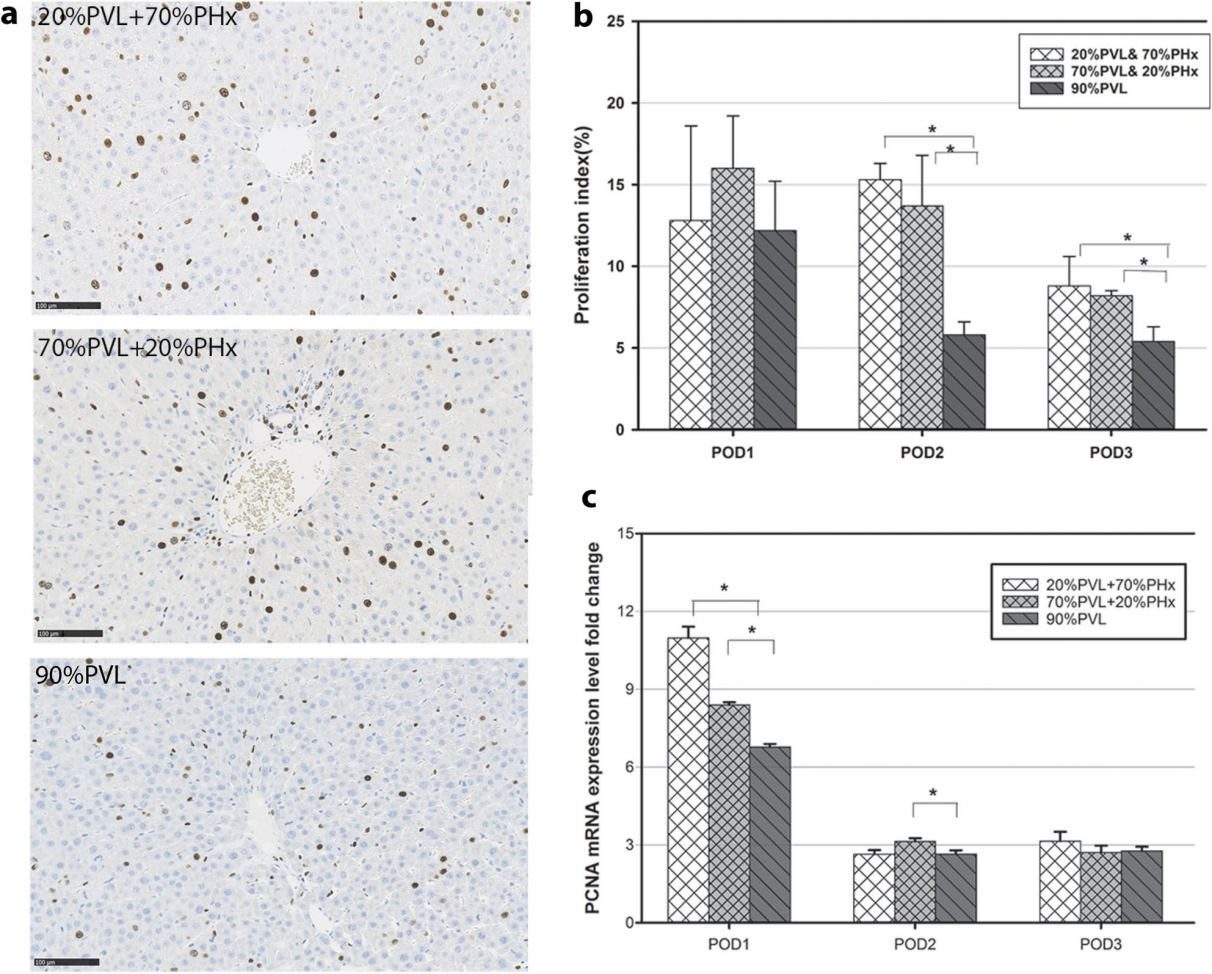
BrdU-staining, hepatocyte proliferation index (PI), PCNA mRNA expression level of the non-ligated FLR. (**a**) Immunohistochemistcal analysis of BrdU incorporation of non-ligated FLR on POD2. More BrdU-positive cells were observed after simultaneous PVL and PHx compared with PVL alone (Scale marker: 100 μm, magnification: ×200). (**b**) Proliferation index (PI) in the non-ligated FLR was significantly higher on POD2 after additional large PHx and additional small PHx compared with PVL alone (PI: 15% vs 6%, 12% vs 6%, *p value < 0.05). (**c**) PCNA mRNA expression level in the FLR was substantially increased after additional PHx compared with PVL alone (fold change: 11 vs 8.4 vs 6.7, *p < 0.05).

Third, we studied hepatocyte proliferation. Hepatocyte proliferation rate in the FLR was significantly higher on POD2 and 3 after simultaneous PVL and PHx compared with 90%PVL without resection. As shown in Fig. 3a, hepatocyte proliferation in FLR on POD2 increased to 15% and 12% after 20%PVL + 70%PHx respectively 70%PVL + 20%PHx, which were significantly higher compared to 90%PVL alone (Fig. 3b). However, there was no statistically significant difference in hepatocyte proliferation rate when comparing small and large additional liver resection.

For further confirmation we assessed PCNA mRNA expression. Here, we observed a significantly higher expression level of PCNA mRNA in the FLR on POD1 after additional large resection compared with the additional small resection and with PVL alone (fold change 11 vs 8.4 vs 6.7, *p < 0.05, Fig. 3c). This effect was not seen on POD2 and 3, where PCNA expression levels in all three groups were about threefold higher compared to normal liver tissue.

### Autophagy induction was observed after simultaneous PVL and PHx

**Figure 4:**
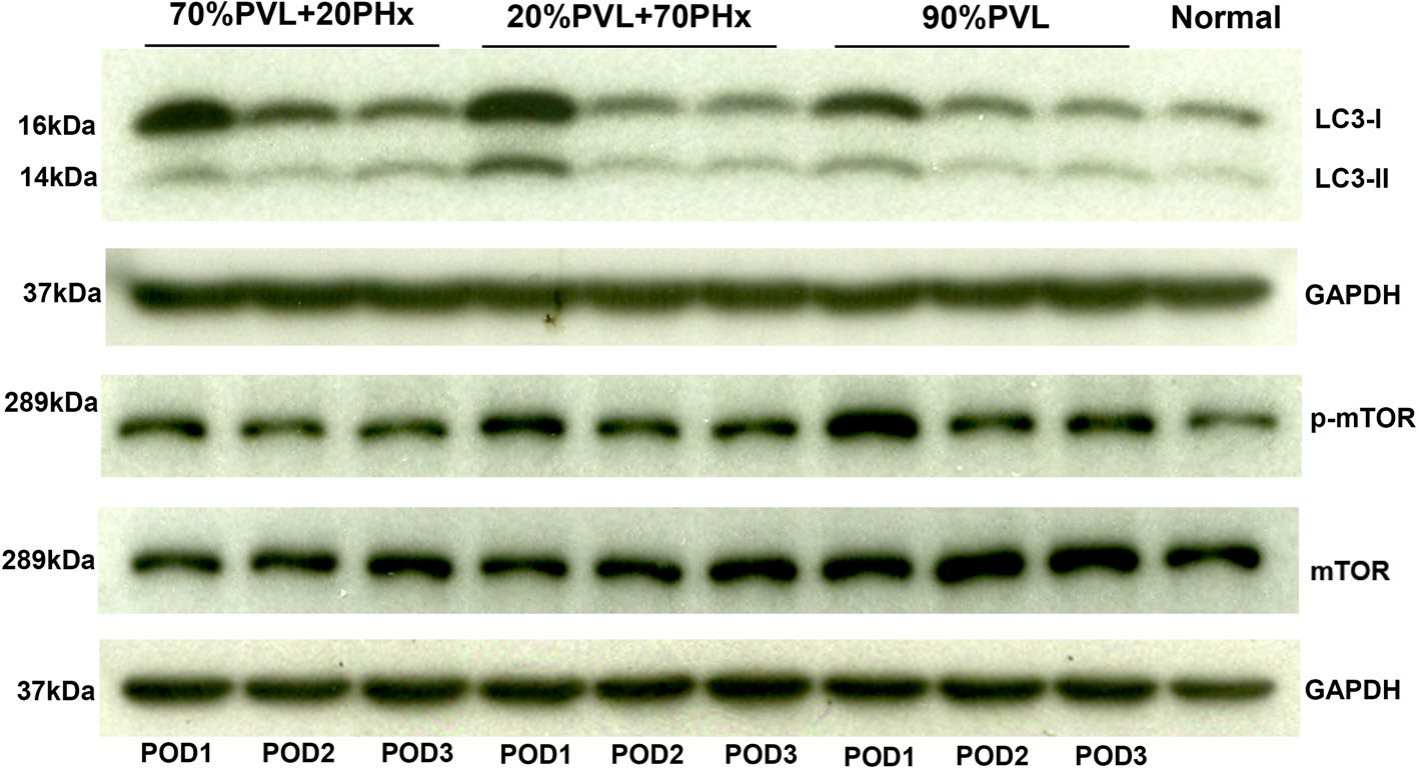
Autophagy related protein expression level in the non-ligated FLR after simultaneous PHx and PVL and PVL alone. Expression level of LC3 and mTOR of total liver homogenates of the FLR was investigated. Substantially higher level of LC3II was observed in additional large PHx and additional small PHx compared with PVL alone. The expression level of p-mTOR showed reverse results.

In the last step, we studied autophagy. LC3 and phosphorylated mTOR (p-mTOR) are well-chracterized markers in scientific community^19,20^. To investigate the role of autophagy in promoting regeneration after simultaneous PVL and PHx, the expression levels of the autophagy related proteins LC3 and p-mTOR were determined (Fig. 4). In the early phase of liver regeneration, protein level of LC3II in the FLR was substantially higher on POD1 after simultaneous PVL and PHx compared with PVL alone, indicating a more pronounced induction of autophagy. Furthermore, the protein levels of p-mTOR in the FLR were higher after PVL alone compared with combined PVL and PHx. These results indicated that simultaneous PVL and PHx induced more autophagy compared to PVL alone.

## Discussion

For patients with large or multiple malignant liver tumors, extended hepatectomy is the most successful strategy resulting in a rather favourable treatment outcome. However, substantial removal of liver mass also causes pronounced hepatic injury and a high risk of liver failure due to inadequate size of the FLR. Major hepatectomy is only considered to be feasible when the FLR is ≥ 40% for patients with cirrhotic liver and ≥ 30% in those with significant steatosis or fibrosis without cirrhosis. In patients with normal liver, a FLR of at least ≥ 20% is needed to sustain hepatic function after major hepatectomy^21^.

Therefore staged liver surgery with or without portal vein occlusion was developed. It is well-known that upon various liver injuries and loss of liver mass, the remaining liver has the ability to compensate the loss of hepatic volume and to restore hepatic function^22,23^. In the past, different strategies were explored to induce regeneration of the FLR (first stage) prior to performing extended liver resection (second stage)^24^. These different strategies have in common that regeneration of the FLR is promoted by inflicting a controlled damage on the future resected liver lobe, resulting in regeneration of the FLR. The damage must be “strong enough” to induce regeneration but “weak enough” not to compromise hepatic function substantially. However, it is unknown which and how much damage should be inflicted on the liver to promote regeneration of the FLR while maintaining sufficient hepatic function during this critical period of time.

Theoretically various types of damage could serve the purpose as long as the damage is restricted to the future resected liver lobes and has little effect on the FLR. Portal vein occlusion including portal vein embolization and portal vein ligation was first established to improve resectability^25^. As outlined before, portal vein occlusion induces atrophy in the ligated liver and promotes hypertrophy in FLR, termed as atrophy/hypertrophy complex (AHC)^26^. The non-ligated liver lobes undergo regeneration to compensate for the loss of hepatic function of the ligated lobes. Restoration of volume and function of the FLR improves the tolerance to the subsequent major hepatectomy performed as second stage operation^27^. The flaw of the procedure is that the waiting time between the two procedures may cause disease progression before proceeding to the second step.

In order to further enhance the regenerative process of the FLR under experimental conditions, several additional surgical strategies were developed. Besides PVL of given liver lobes, surgical modifications such as two-stage PVL, additional bile duct ligation and additional hepatic vein ligation were introduced in different experimental models (Table 1).

**Table 1:** Experimental studies investigating regeneration in non-ligated liver (FLR) after PVL in rat model.

Year	Author	Animal	Surgical treatment	Hypertrophy of FLR		Regeneration of FLR		
				Extent^a^ (%)	Observation time	Parameter	Enhanced regeneration	Observation time
2008	Sugimoto et al.^30^	Wistar rats	90%PVL	315%	POD7	PCNA	+++	POD2
			2-stage 90%PVL^b^	560%	POD14	Mitosis	+++	POD8
2015	Ren et al.^31^	SD rats	90%PVL	250%	POD7	Ki67	++	POD3
			90%PBL^c^	310%	POD7		+++	POD3
2019	Kawaguchi et al.^33^	Wistar rats	90%PVL	393%	POD7	Ki67	++	POD1
			90%PVL + 30%HVL	777%	POD7		+++	POD3
2019	Jia et al.^37^	SD rats	70%PVL	291%	POD7	Cyclin D1	+++	POD2

*SD rats* Sprague–Dawley rats, *HVL* hepatic vein ligation, + mild increase, ++ moderate increase, +++ substantial increase.

^a^Weight ratio of the non-ligated liver lobe to pre-operative value.

^b^2-stage PVL: first stage: 70%PVL, second stage: 20%PVL on POD7.

^c^PBL: simultaneous portal vein ligation and bile duct ligation.

Two-stage PVL, 70%PVL followed by the second 20%PVL seven days later, was established by Sugimoto et al. This new strategy was proven to induce more hypertrophy in the FLR compared to the conventional one-stage PVL^30^. Ren et al. performed simultaneous bile duct and portal vein ligation in a rat model. They demonstrated that the simultaneous procedure accelerated the AHC and promoted hepatic proliferation in the FLR compared to PVL alone^31^. Additional hepatic vein ligation at the time of PVL was introduced by Kawaguchi et al. They found that additional hepatic vein occlusion resulted in more damage in the ligated lobe and induced more regeneration in the non-ligated liver compared with PVL alone^33^.

In the present study, we suggest not to induce additional damage on the portally deprived liver but to remove part of the liver, as done clinically when “cleaning” the liver from bilobar metastasis. The additional resection serves two purposes: on the one hand to enhance regeneration of the FLR and on the other hand to mimic the clinical situation of “reducing the tumor load” of the liver. Furthermore using our novel model combining different extent of PVL with different extent of PHx, we can study intrahepatic size regulation. In the previous study^18^, we demonstrated that simultaneous PVL and PHx was a well-tolerated procedure allowing all rats to survive even when reducing the size of the healthy FLR to only 10% of the original liver mass. In the present study, we found that the combined procedure induced more hepatocyte proliferation and faster course of liver hypertrophy in the FLR than after PVL alone. However, we did not see a statistically significant additional effect on size regulation of the FLR when increasing the size of resection.

Using our model of simultaneous PVL and PHx, we observed previously in the ligated lobe that induction of the energy consuming process of hepatocyte proliferation was related to an increase in the energy providing process autophagy^18^. In the present study, we observed that increased regeneration of the non-ligated lobe after additional resection was also associated with an increased expression of autophagy related proteins.

The relationship between autophagy and liver regeneration is currently discussed in many experimental studies (see Table 2). Recently, experimental evidence is accumulating that autophagy activity plays an essential role in promoting regeneration and reducing organ damage^32,34–36^. It was reported that augmenter of liver regeneration promoted hepatocyte proliferation in mice through activation of autophagy in CCl_4_-induced acute liver injury^32^. In a mouse model with 2/3 hepatectomy, Lu et al. found that miR-1907 accelerated hepatocyte proliferation via activating autophagy. They further observed a significant decrease in miR-1907-induced liver regeneration after inhibiting autophagy^36^. Activation of autophagy in the non-ligated lobe following 70%PVL was also observed in a rat model. The study suggested that the increased autophagy activity was positively related to rapid hepatocyte proliferation^37^. Our present study further confirms these observations, since we also found that simultaneous PVL and PHx induced not only more regeneration but also more autophagy in the FLR than PVL alone.

**Table 2:** Experimental studies investigating the protective role of autophagy during liver regeneration.

Year	Author	Experimental model	Surgical strategy	Pharmacological intervention	Autophagy		Regeneration	
					Parameter	Observation time	Parameter	Observation time
2014	Toshima et al.^35^	Wild-type Atg5 Mice	70%PHx	/	LC3-II++, P62+	POD1	BrdU+++, Cyclin D+++	POD1
		L-Atg5KO mice^a^	70%PHx	/	LC3-II−−−, P62+++	POD1	BrdU+, Cyclin D−	POD1
		Hepatocytes	/	HGF^b^	LC3-II+++	/	Cyclin D+++	/
2015	Lin et al.^34^	C57BL/6 Mice	70%PHx	/	LC3-II++, P62+	POD1	Ki67++, PCNA++	POD2
				Amiodarone	LC3-II+++, P62−	POD1	Ki67+++, PCNA+++	POD2
				Chloroquine	LC3-II+++, P62+++	POD1	Ki67+, PCNA−	POD2
2016	Shi et al.^32^	BALB/c mice	/	CCl_4_	LC3-II+++, P62+++	POD2	PCNA−−−, CyclinD−−−	POD2
				CCl_4_ + ALR	LC3-II+++, P62−−−	POD2	PCNA+++, Cyclin D+++	POD2
				CCl_4_ + 3-MA^c^	/	/	PCNA−−−, CyclinD−−−	POD2
2018	Lu et al.^36^	C57BL/6 Mice	70%PHx	miR-1907	LC3-II+++, P62−−−	POD2	BrdU+++, PCNA+++	POD2
2019	Jia et al.^37^	SD rats	70%PVL	/	LC3-II+++	POD1	Cyclin D+++	POD1
2019	Matsumoto et al.^39^	C57BL/KsJ m + /m + mice(control)	70%PHx	/	LC3-II+++, P62-	POD1	PCNA+++, Cyclin D+++	POD2
		C57BL/KsJ db/db mice^d^	70%PHx	/	LC3-II+++, P62+++	POD1	PCNA+, Cyclin D−	POD2

^a^Atg5-deficient mice.

^b^Hepatocyte growth factor.

^c^3-MA: 3-Methyladenine, autophagy inhibitor.

^d^Model of liver steatosis and diabetes.

Based on these recent studies about the critical role of autophagy in liver regeneration, we suggest that the process of regeneration in the FLR after simultaneous PHx and PVL or PVL alone might also be further augmented by applying autophagy inducers. Application of an mTOR-independent autophagy promoter in an animal model of PVL or simultaneous PVL and PHx seems to be an interesting approach to be investigated. This strategy might induce more proliferation and augment hypertrophy of the FLR more than PVL alone, thereby shortening the interval time needed for restoring the original liver mass.

In summary, better understanding of the interaction between liver regeneration and autophagy after PVL and PHx is useful to refine therapeutic strategies for patients with primarily irresectable liver disease. Additional PHx will not only promote regeneration of the FLR but also reduce the tumor load in the first step, thereby preventing the increase of tumor burden during the waiting period.

## Conclusion

Simultaneous PVL and PHx procedures were performed on healthy rats without malignant disease resulting in augmented regeneration of the FLR and an enhancement of autophagy. These observations call for further exploration of promoting autophagy as novel strategy to augment liver regeneration in this complex surgical model.

## Methods

### Animals

Male Lewis rats weighing 250–300 g (9–10 weeks old), purchased from Charles River, Sulzfeld, Germany, were used in the present study. The rats were housed under constant room temperature and humidity and a 12 h-light–dark cycle in a conventional animal facility. Water and rat chow were provided ad libitum.

### Ethics statement

The protocols were approved by the Thüringer Landesamt für Verbraucherschutz, Thuringia, Germany (Approval-Number: 02-024/13). All experiments and housing of animals were performed in compliance with the current German regulations and guidelines for animal welfare and the ARRIVE Guidelines for Reporting Animal Research^28^.

### Experimental design

**Figure 5:**
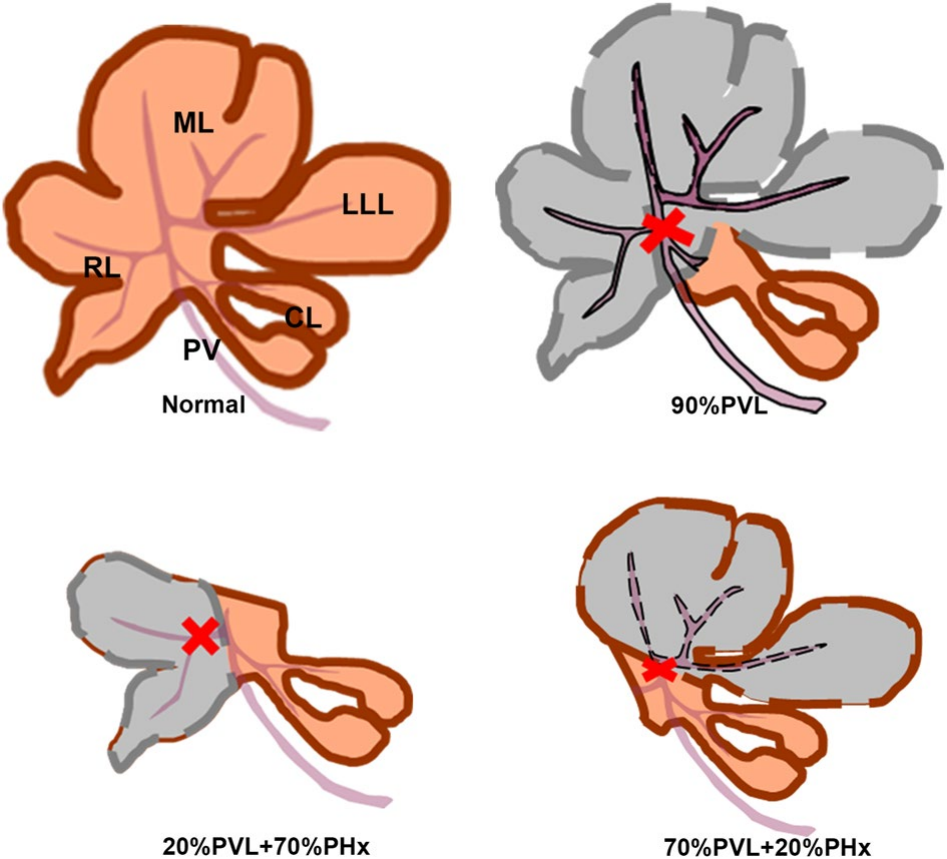
Sketches of experimental groups. *LLL* left lateral lobe, *ML* median lobe, *RL* right lobe, *CL* caudate lobe, *PV* portal vein; liver lobe with grey color: ligated lobe, liver lobe with orange color: non-ligated FLR, red cross: portal vein ligation.

Male Lewis rats were assigned into two experimental groups (Fig. 5): 20%PVL + 70%PHx: ligation of right portal vein (20% of the liver mass) followed by resection of left lateral and median lobes (70% of the liver mass); 70%PVL + 20%PHx: ligation of right and left portal vein followed resection of right lobe. The control consisted of additional twenty-four rats subjected to 90% PVL group which underwent ligation of left lateral, median and right lobes portal vein. Animals were observed for 24 h, 48 h, 72 h and 7 days postoperatively (n = 6/observation time point).

### Operative procedures and postoperative management

The rats were acclimatized for 1 week before operation. Surgical procedures were conducted under inhalation anesthesia consisting of a mixture of 3% isoflurane and pure oxygen at a flow rate of 0.5 L/min (isoflurane vaporizer, Sigma Delta, UK). Following skin disinfection, the abdominal cavity was opened via a transverse upper abdominal incision. The intestine was eventerated and covered by wet gauze. Portal vein branches were dissected from artery and bile duct and then ligated with 6–0 prolene suture using an operating microscope (Zeiss, magnification 10–25**×**, Germany). Ligation of right portal vein represented 20%PVL, ligation of left portal vein represented 70%PVL, and ligation of both right and left portal vein represented 90%PVL. Additional PHx was then performed as described previously^17^. A Mosquito-clamp was placed on each liver lobe 2–3 mm distal from the inferior vena cava. The clamp was kept stable while removing the liver lobe and placing 2–4 piercing sutures. Next, the clamp was removed followed by replacing the abdominal viscera and closing abdomen. A dose of 0.05 mg/kg body weight of buprenorphine was applied as analgesic treatment to all animals postoperatively (Temgesic, Essex Pharma GmbH, Germany). Daily evaluation of general condition and activity was carried out after operation.

### Harvesting and sampling

Rats were sacrificed postoperatively day 1, 2, 3 and 7. For detecting hepatocyte proliferation in the FLR, rats were injected intravenously with a single dose of 50 mg/kg 5-bromo-2-deoxyuridine (BrdU, SIGMA-ALDRICH, St. Louis, USA) one hour before sacrifice.

Blood collection was performed under anesthesia and liver tissue was harvested. The wet weight of remnant liver was measured and the liver weight/body weight ratio was calculated using the following formula: individual liver lobe weight (g)/body weight (g) × 100%.

Serum was isolated from the blood. The serum aspartate aminotransferase (AST) and alanine aminotransferase (ALT) were measured by using the AEROSET System (Abbott Laboratories, Wiesbaden, Germany).

### Histology and immunohistochemistry (IHC) analysis

Liver tissue was fixed by immersion in 4.5% buffered formalin for 48 h and embedded in paraffin. Paraffin sections of 4 µm thickness were prepared. BrdU-staining was performed for visualization of hepatocyte proliferation. A monoclonal anti-BrdU antibody (Dako, Hamburg, Germany) was use in the staining, following the protocol described previously^17^. Immunohistochemistry results were analyzed with the Histokat software (Fraunhofer MEVIS, Bremen, Germany) using a nuclei detection algorithm based on a previously published image analysis method^17^. The algorithm used machine learning techniques to recognize BrdU-positive and BrdU-negative nuclei, taking into account color, roundness and size features. The proliferation index was calculated as the fraction of the amount of BrdU-positive nuclei to the total number of hepatocyte nuclei, according to previously reported protocol.

### Protein extraction and Western blotting

Liver tissues were homogenized in the RIPA buffer (sigma, R0278) containing the Protease and phosphatase inhibitor cocktail (Thermo Scientific, USA). The concentration of total proteins was measured by using BCA protein assay kit (Thermo Scientific, USA) and ELISA reader device. Equal amounts of protein were denatured with Laemmli sample buffer (Bio-Rad, USA). Proteins were separated in electrophoresis process and transferred to the polyvinylidene difluoride membranes. The membranes were washed and blocked as previously reported^38^. The blots were cut prior to hybridization with primary antibodies. Primary antibodies rabbit anti-light chain 3 (LC3; 1:1000, Cell signaling Technology), rabbit anti-mammalian target of rapamycin (mTOR, 1:1000,Cell signaling Technology), rabbit anti-phospho-mTOR (Ser2448, 1:1000, Cell signaling Technology) and rabbit anti-glyceraldehyde-3-phosphate dehydrogenase (GAPDH; 1:10,000, Cell signaling Technology) were applied to the membranes and were incubated at 4 °C overnight. After the membranes being washed, the second antibody (Goat polyclonal antibody to rabbit IgG; 1:5000) was then used. After being probed by using enhanced chemiluminescence western blotting substrate (GE Healthcare), the signals were visualized by using Fusion FX7 (Labtech International Ltd, Heathfield, United Kingdom). All western blots were repeated 3 times (Supplementary Information).

### Real time PCR analysis

Total RNA was isolated from liver tissue sections using the RNeasy kit (Qiagen, Hilden, Germany) following the manufacture’s instruction. RNA samples were reverse transcribed to cDNA by using the First-Strand cDNA synthesis KIT (Invitrogen, Carlsbad, USA). The mRNA expression of PCNA was investigated using the Brilliant probe-based QPCR Mater Mix kit (Agilent, Santa Clara, USA), performed by using M3000P QPCR System (Stratagene, La Jolla, USA). The mRNA level of HPRT was served as an endogenous control. The primers (eurofins Genomics, Germany) were listed as following: proliferating cell nuclear antigen (PCNA):forward5′-TGAACTTTTTCACAAAAGCCACT-3′, reverse5′TGTCCCATGTCAGCAATTTTA-3′; hypoxanthine guanine phosphoribosyltransferase (HPRT): forward5′-GACCGGTTCTGTCATGTCG-3′, reverse5′-ACCTGGTTCATCATCACTAATCAC-3′. Relative fold of gene expression of samples was calculated by the well-accepted 2^–∆∆Ct^ method.

### Statistical analysis

SigmaPlot 13.0 (Statcon, Witzenhausen, Germany) was adopted for data analysis. The differences between groups were compared using the one way independent ANOVA test. Statistical differences were considered significant when *p* values were less than 0.05.

## Supplementary Information

Supplementary Information.

## Data Availability

All data generated or analyzed during this study are included in this presented article. Parts of the data in this manuscript were presented in our previous paper^18^ and Dr. Wei’s thesis. (https://www.dbthueringen.de/servlets/MCRFileNodeServlet/dbt_derivate_00039437/disswei.pdf).

## Abbreviations

PHx: Partial hepatectomy
PVL: Portal vein ligation
PVE: Portal vein embolization
AST: Aspartate aminotransferase
ALT: Alanine aminotransferase
BrdU: 5-Bromo-2-deoxyuridine
PI: Proliferation index
POD: Postoperative day
PCNA: Proliferating cell nuclear antigen
