# Supplementary material for: Additional partial hepatectomy at the time of portal vein ligation accelerates the regeneration of the future liver remnant
Source: Sci Rep. 2021 Jun 3;11:11740. doi: 10.1038/s41598-021-90819-x (PMC8175446; doi:10.1038/s41598-021-90819-x)

Additional partial hepatectomy at the time of portal vein ligation accelerates the regeneration of the future liver remnant

Chuanfeng Hua, *MS* [a]* Weiwei Wei, *MD* [a]* Tianjiao Zhang, *MD* [b] Fengming Xu, MS[a] Olaf Dirsch, *PhD* [c] André Homeyer, *PhD* [d] Utz Settmacher, *MD* [a] Uta Dahmen, *MD*[a#]

[a] Department of General, Visceral and Vascular Surgery, Jena University Hospital, Jena, Germany

[b] Department of Radiotherapy and Radiation Oncology, Jena University Hospital, Jena, Germany

[c] Institute of Pathology, Klinikum Chemnitz gGmbH, Chemnitz, Germany

[d] Fraunhofer Institute for Digital Medicine MEVIS, Bremen, Germany

* These authors contributed equally to this work.

# Corresponding author

E-mail: Uta.Dahmen@med.uni-jena.de Tel.: 03641-9325350 Fax: 03641-9325352

Article type: Original research

Supplementary information for blots in **Figure 4**

Blots order

Blots	1	2	3	4	5	6	7	8	9	10
Experiment groups	70%PVL+20%PHx			20%PVL+70%PHx			90%PVL			Normal
Observation time	POD1	POD2	POD3	POD1	POD2	POD3	POD1	POD2	POD3	-

Supplementary figure 1. LC3_cropped part shown in Figure 4

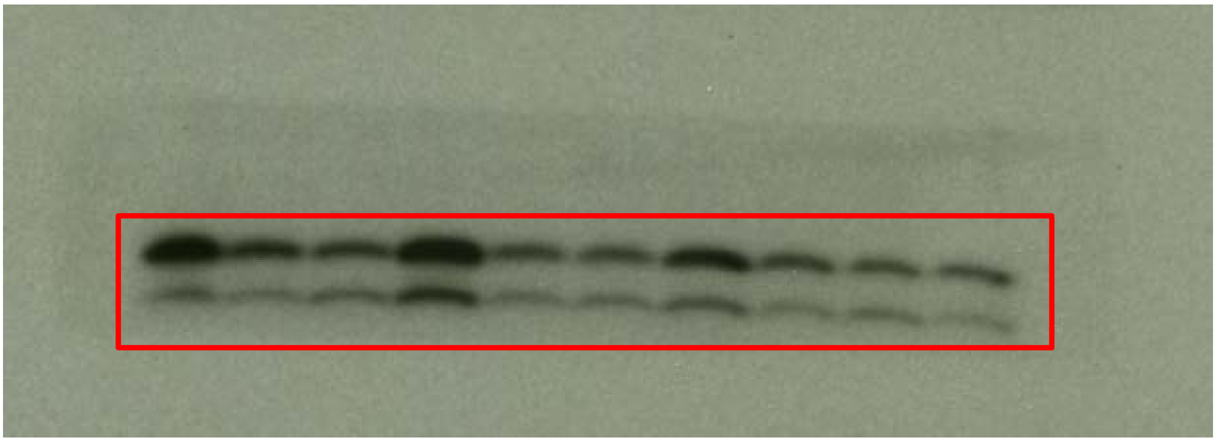

Supplementary figure 2. GAPDH of LC3_ cropped part shown in Figure 4

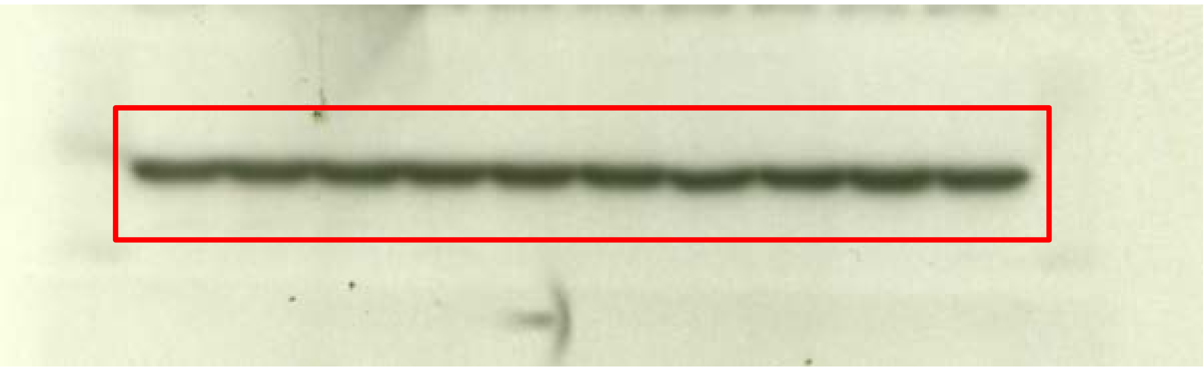

Supplementary figure 3. p-mTOR and mTOR_cropped part shown in Figure 4

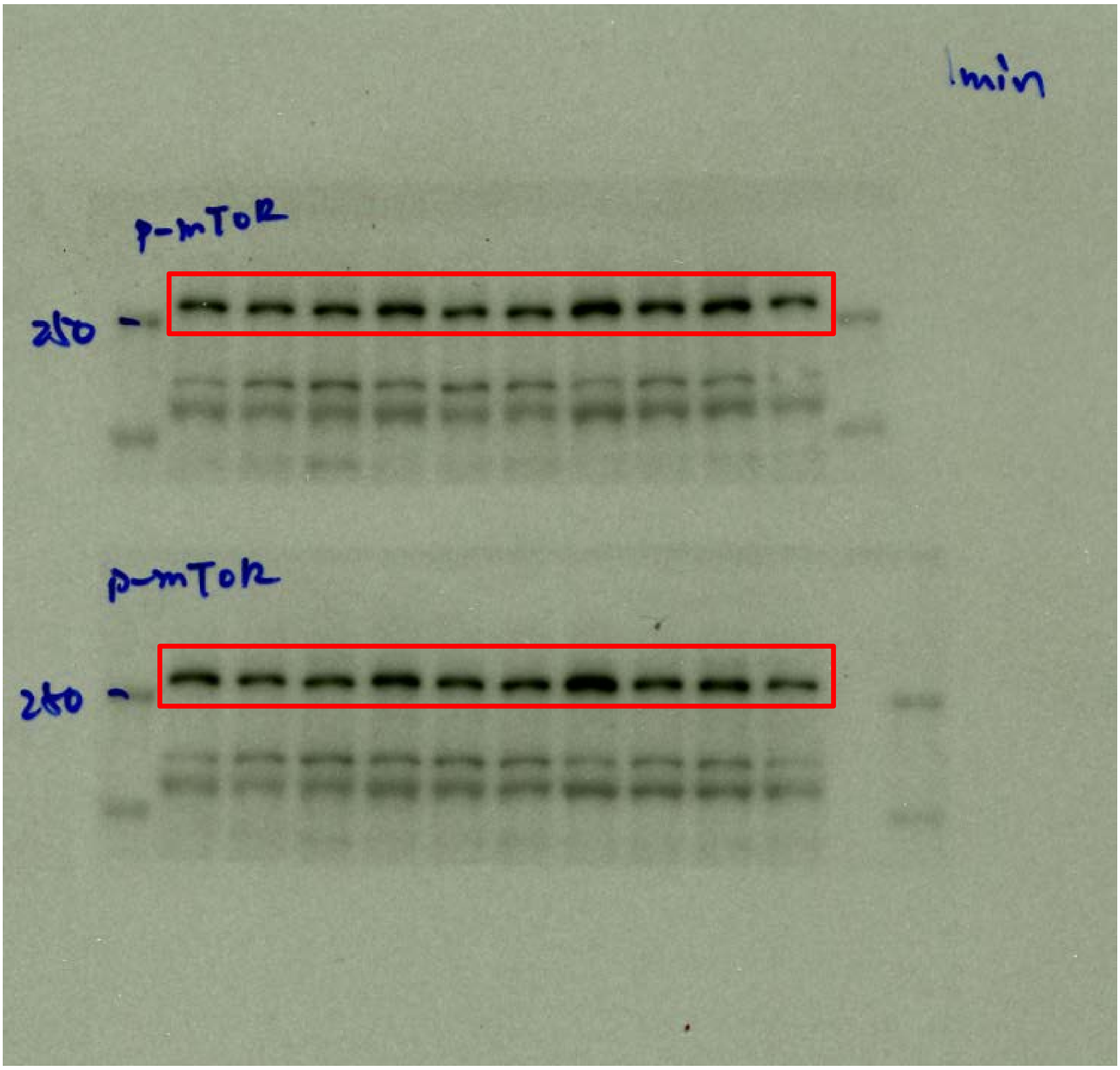

Supplementary figure 4. GAPDH of mTOR and p-mTOR_ cropped part shown in Figure 4

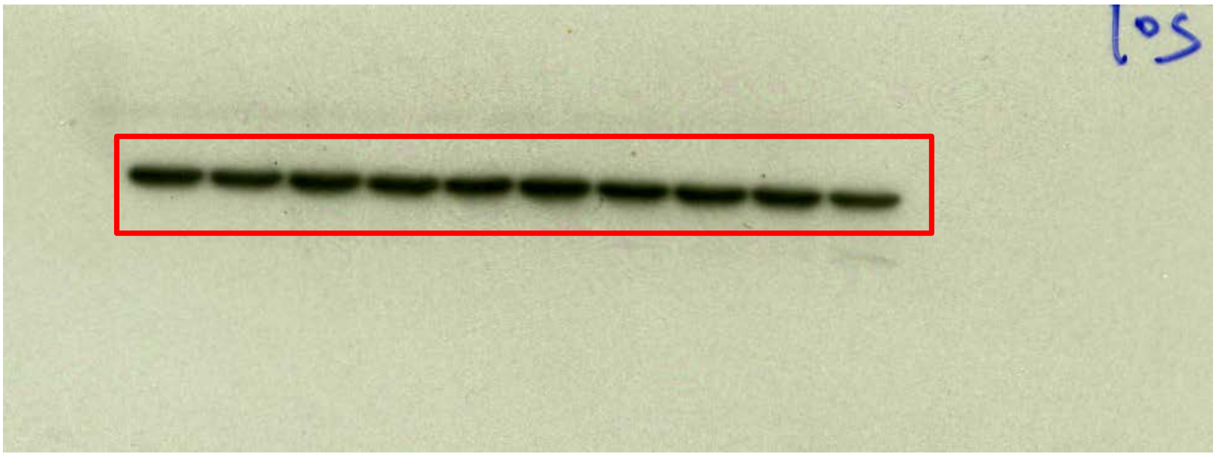

Replicates of blots_not shown in the manuscript

Supplementary figure 5. LC3 replicate2

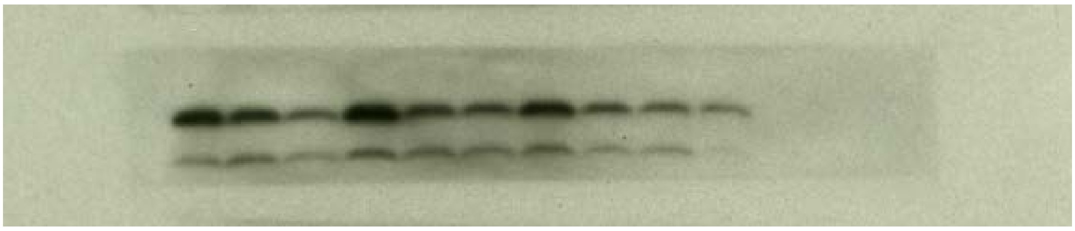

Supplementary figure 6. LC3 replicate3

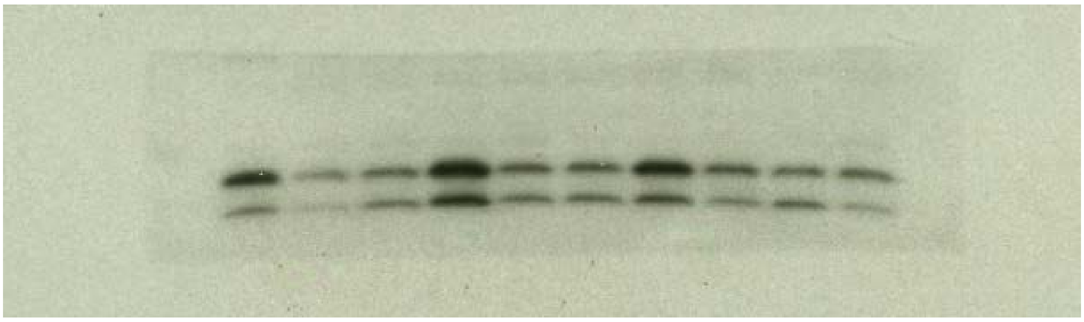

Supplementary figure7. mTOR and p-mTOR replicate2

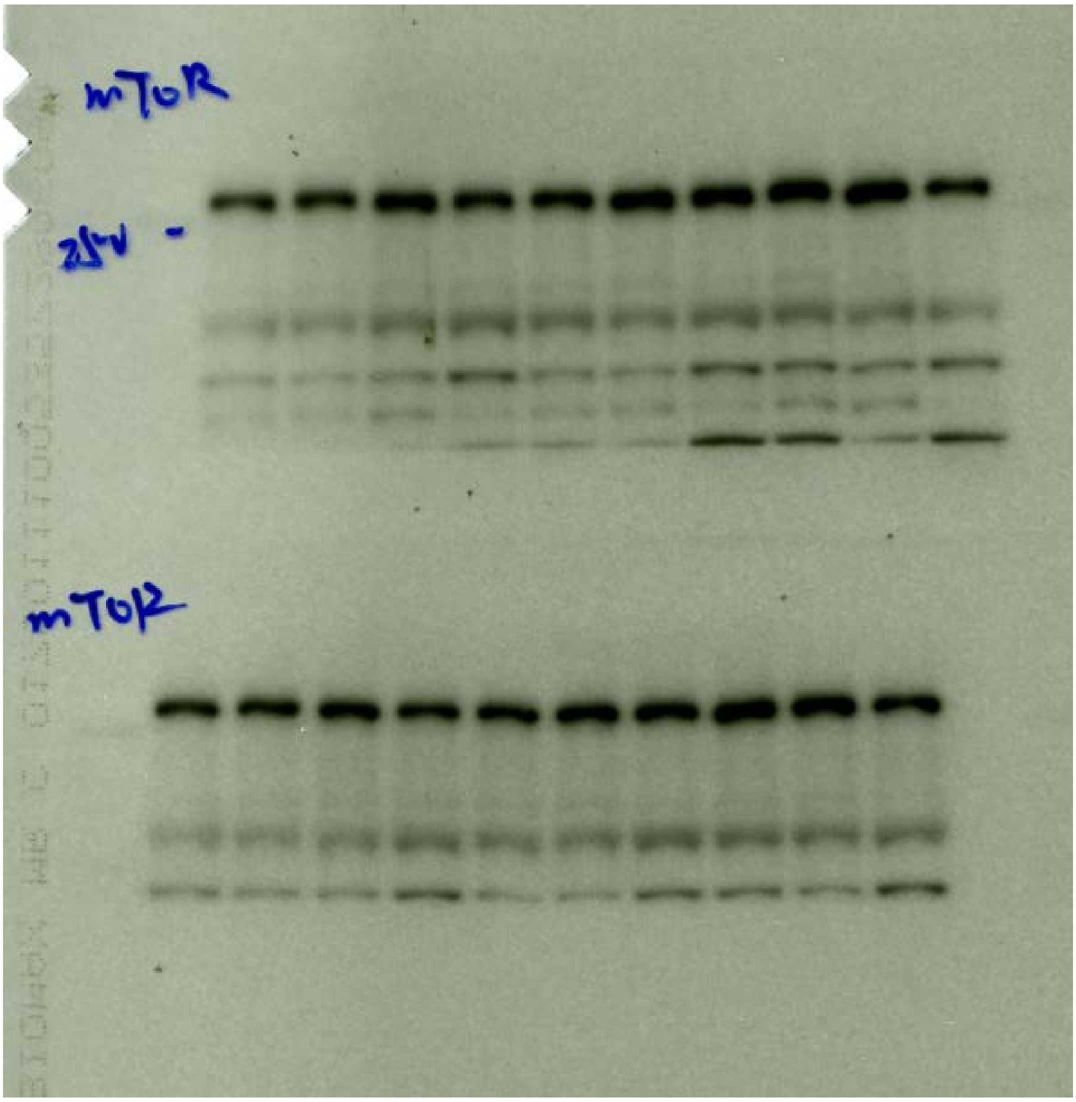

Supplementary figure 8. mTOR replicate3

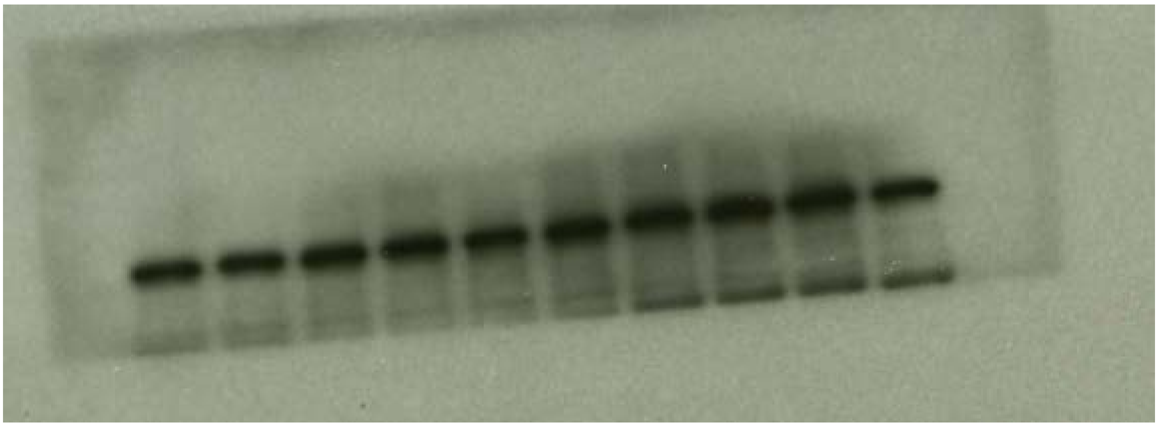

Supplementary figure 9. P-mTOR replicate2

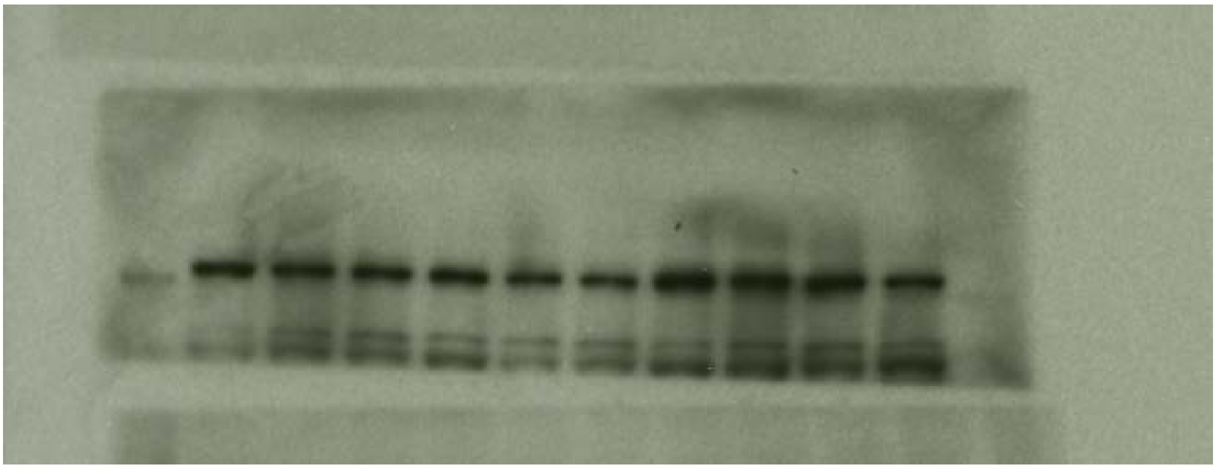

Supplement: Supplementary file 1 — Supplementary Information. [file 41598_2021_90819_MOESM1_ESM.pdf]
